# Supplementary material for: Alternative Polyadenylation Characterizes Epithelial and Fibroblast Phenotypic Heterogeneity in Pancreatic Ductal Adenocarcinoma
Source: Cancers (Basel). 2024 Feb 2;16(3):640. doi: 10.3390/cancers16030640 (PMC10854489; doi:10.3390/cancers16030640)
Supplement: Supplementary file 1 [file cancers-16-00640-s001.zip › cancers-2819363-supplementary.pdf]

Venkat S and Feigin ME., Figure S1

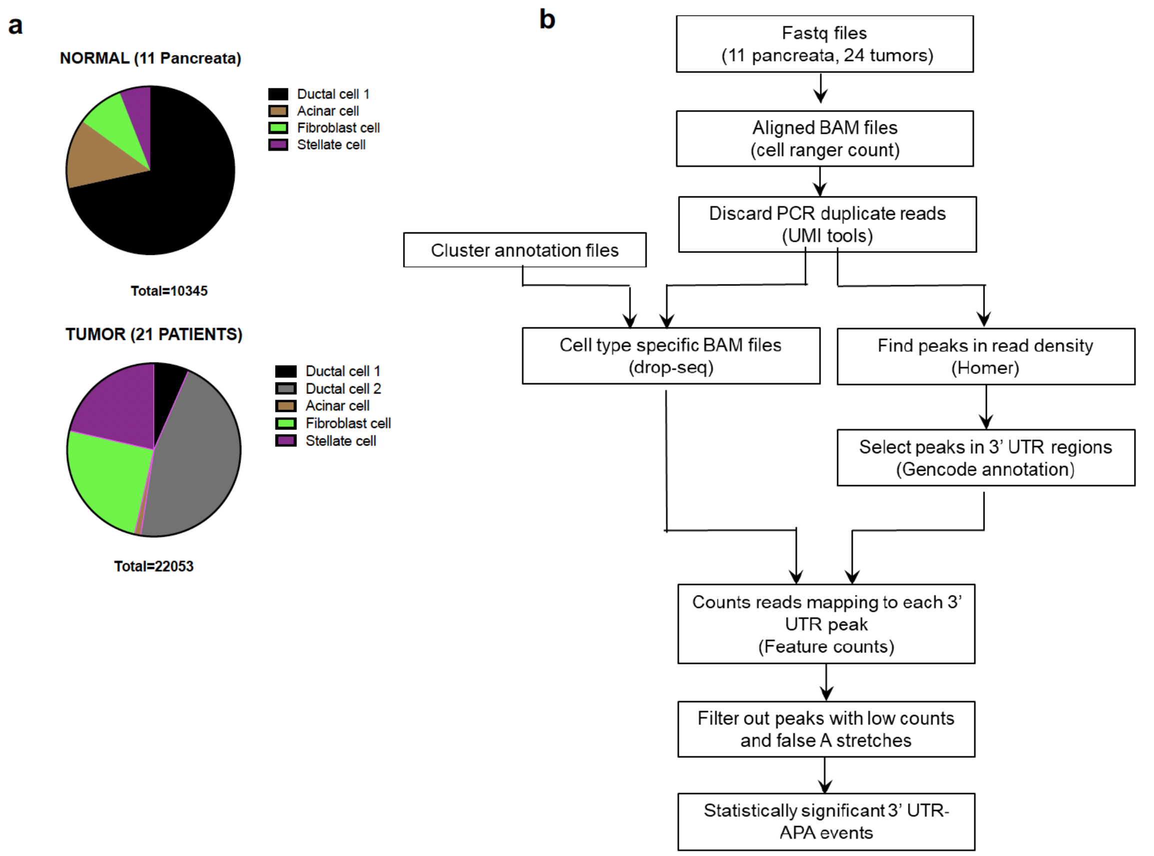

Figure S1. Description of the scRNA-seq dataset and the workflow used to quantify 3′ UTR-APA in PDAC. S1a. A pie graph representing the single cell dataset that was used for downstream analyses of 3′ UTR-APA patterns. Proportion of ductal and acinar cells in the epithelium, and fibroblast and stellate cells in the stroma are highlighted.
S1b. The workflow implemented to detect and quantify 3′ UTR-APA events from single cell sequencing data (adapted from [18]).

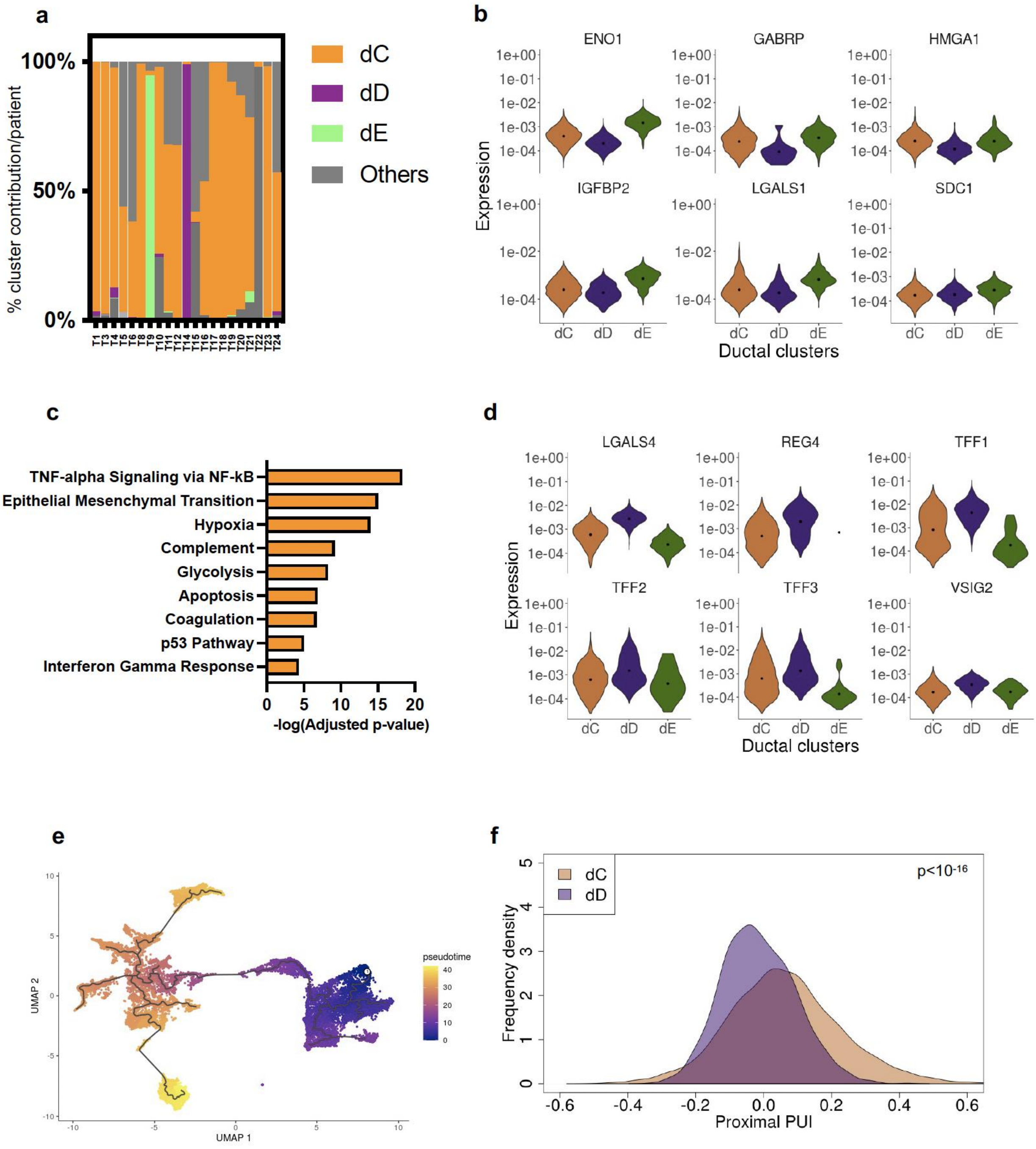

Figure S2. Proximal APA in tumor epithelium is associated with PDAC progression and malignant phenotypes.

S2a. Barplot showing contribution of different ductal subclusters to each PDAC patient.

S2b. Violin plots of select metastatic markers across ductal cell type 2 subclusters ($p<0.001$).

S2c. Significant enriched pathways (FDR < 0.01) associated with genes overexpressed in dE compared to dC.

S2d. Violin plots of select well-differentiated PDAC markers across ductal cell type 2 subclusters ($p<0.001$).

S2e. Pseudo-time analysis depicting progression of ductal cell states (purple, early; yellow, late) based on their gene expression profiles.

S2f. Distribution of mean proximal PUI of single cells in subcluster dD (purple) compared to subcluster dC (brown) ($p<10^{-16}$).

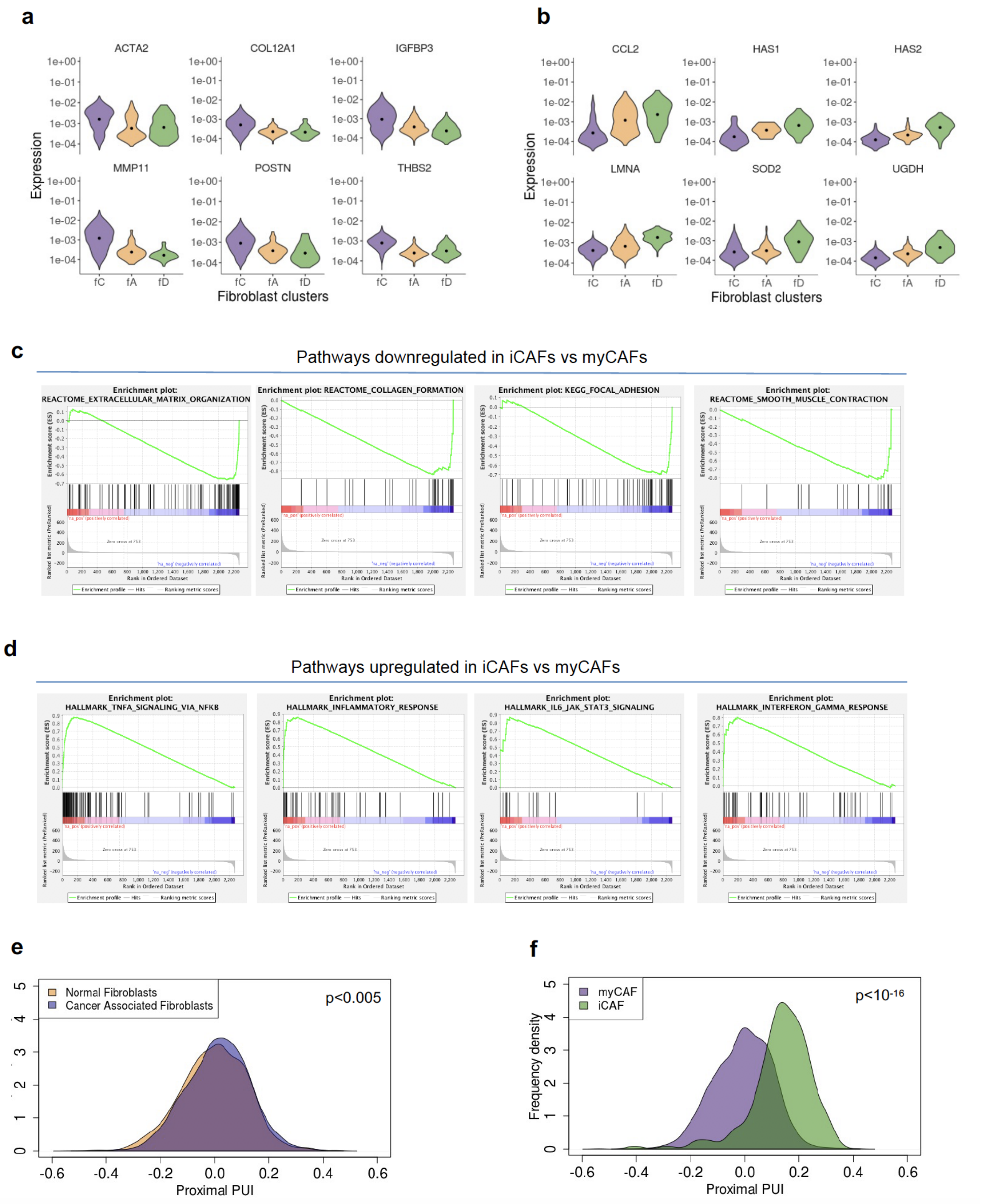

Figure S3. Increased proximal APA characterizes the inflammatory CAF phenotype.
S3a. Violin plots of myCAF markers across normal fibroblasts (fA, orange) and specific tumor fibroblast subclusters (fC, purple; fD, green).
S3b. Violin plots of iCAF markers across normal fibroblast (fA) and specific tumor fibroblast subclusters (fC, fD).
S3c. GSEA of significantly downregulated pathways in iCAFs compared to my CAFs.
S3d. GSEA of significantly upregulated pathways in iCAFs compared to my CAFs.
S3e. Distribution of mean proximal PUI of single cells of normal fibroblasts (orange) compared to tumor fibroblasts (blue) ($p<0.005$).
S3f. Distribution of mean proximal PUI of single cells of iCAFs (green) compared to myCAFs (purple) ($p<10^{-16}$).
